# A Systematic Review of Work Organization, Work Environment, and Employment Conditions in Warehousing in Relation to Gender and Race/Ethnicity

**DOI:** 10.1093/annweh/wxac098

**Published:** 2023-01-30

**Authors:** Klara Rydström, Jennie Jackson, Kristina Johansson, Svend Erik Mathiassen

**Affiliations:** Department of Social Sciences, Technology and Arts, Luleå University of Technology, Laboratorievägen 14, 971 87 Luleå, Sweden; Centre for Musculoskeletal Research, Department of Occupational Health Sciences and Psychology, University of Gävle, 801 76 Gävle, Sweden; Department of Social Sciences, Technology and Arts, Luleå University of Technology, Laboratorievägen 14, 971 87 Luleå, Sweden; Centre for Musculoskeletal Research, Department of Occupational Health Sciences and Psychology, University of Gävle, 801 76 Gävle, Sweden

**Keywords:** distribution center, fulfillment center, inequality regimes, occupational health, working conditions

## Abstract

**Objectives:**

Studies in the goods supply chain in areas outside of warehousing show evidence of gender and racial/ethnic inequalities in working conditions (i.e. in work organization, work environment, and employment conditions). This review aimed to identify, summarize, and discuss research focused on inequality in warehousing and its effects on warehouse working conditions. In the review, racial/ethnic inequality includes inequality related to country of birth and (im)migration status.

**Methods:**

We performed a systematic search in the Scopus and Web of Science databases to identify warehouse studies that addressed working conditions and (in)equality at a workplace level. Screening of records was performed using the Rayyan systematic review tool. Risk of bias was assessed according to established methods and checklists.

**Results:**

Database searches yielded 4910 articles. After title-abstract-keyword and full-text screenings, 21 articles were included. Results showed inequality based on gender and race/ethnicity in both work organization (different tasks were performed by different groups of employees), work environment conditions (physical and psychosocial aspects differed), and employment conditions (disparate employment types and incomes between groups of employees). Health differences, as a possible result of unequal working conditions, were evident between different racial/ethnic groups of employees. A hierarchy that included both gender and race/ethnicity was found, with (im)migrant and racialized women positioned at the bottom.

**Conclusions:**

We found evidence that gender and race/ethnicity influenced work organization, work environment conditions, and employment conditions. Evidence was found for an intersection between gender and race/ethnicity. To improve working conditions, and subsequently occupational health, we encourage researchers to simultaneously consider gender and race/ethnicity factors at work, and to consider both why inequality is present and how it impacts working conditions in future studies of warehousing, particularly in online retailing.

**What’s Important About This Paper?**

Warehouses play a key role in the global economy and warehouse workers make an essential part of the supply chain. This systematic review is important because it identifies, summarizes, and discusses research focused on inequality in warehousing and its effects on warehouse working conditions. Evidence that gender and race/ethnicity influenced work organization, work environment conditions, and employment conditions suggests gender and race/ethnicity are mutually constitutive factors shaping outcomes of inequality. The results brought forward by this review are useful for researchers and policy makers working to improve working conditions, and subsequently occupational health, in warehousing.

## Introduction

Warehouses play a key role in the global economy. The significance of warehouses in the supply chain began increasing at the start of the logistics revolution in the 1970s and has grown in parallel with increasing customer power and influence (Bonacich and Wilson, 2008). Warehouses and the work performed there have thus become a decisive factor for the efficiency and success of manufacturers and retailers (De Koster *et al.*, 2007; Bonacich and Wilson, 2008).

In general, the process of handling goods in warehouses can be divided into unloading and unpacking of incoming goods; sorting and allocation of goods to shelves; order picking; order packing; and palletizing of outgoing goods. Order picking is generally the most labor-intensive part of the process (Bartholdi and Hackman, 2014). Manual materials handling (MMH) in picking is associated with a relatively high occurrence of musculoskeletal disorders (MSDs) among warehouse workers, most notably in the lower back and wrists (Marras *et al.*, 1999; Basahel, 2015). The type of goods and their placement on the shelves are key workplace ergonomic factors that determine the physical loads on the worker (Lavender *et al.*, 2010). A study in the order picking area of a warehouse showed that physical risks for MMH workers placed there in the work organization could be decreased through workplace design, including consideration of shelf placement of goods and zoning of goods sections (Otto *et al.*, 2017; cf. Kourinka *et al.*, 1994). The risk of MSDs was higher among those performing MMH warehouse work compared to those driving forklifts (Gajšek *et al.*, 2020), even though workers using assisting technology, such as forklifts, have been shown to have poor working postures (Braam *et al.*, 1996).

In addition to work organization and work environment conditions, employment conditions have been shown to be an important factor to occupational health in warehouse workers. Studies show that the use of temporary agency workers is common in warehousing (Bonacich and Wilson, 2008; Allen, 2010; Struna *et al.*, 2012). This is partly explained by the ‘just-in-time’ production approach often used in warehousing, in which workforce flexibility is a means for employers to keep efficient supply chains and avoid unmotivated costs. Temporary agency workers are in a precarious position compared to standard permanent employees regarding (lack of) long-term contracts and benefits, which may negatively affect their experiences of work and well-being (Bonacich and Wilson, 2008; Allen, 2010; Struna *et al.*, 2012). Taken together, the warehouse literature makes visible that both work organization, work environment conditions, and employment conditions are relevant aspects to consider in the studies of this sector.

Moreover, it is likely that not all workers are treated equally in warehousing. In this review, we use *inequality* to refer to ‘systematic disparities between participants’ (Acker, 2006) associated with gender, race, ­ethnicity, country of birth, and (im)migration status of the employees. Systematic disparities can manifest at all levels of the workplace, e.g. when a group of workers is a priori deemed most suitable for a task and preferentially targeted in recruitment- and promotion practices, in wage negotiations, and in informal interactions between employers and employees. Practices and processes that create inequality also tend to be reinforced on a symbolic level and in the construction of images of masculinity and femininity, work tasks, and skills (Acker, 2006). Whilst we acknowledge that race, ethnicity, country of birth, and (im)migration status are differentiated both in theory and lived experiences (cf. Hübinette and Mählck, 2015: 1), we have, in the present review, grouped these aspects together under the single term ‘race/ethnicity’. Our use of the ‘race/ethnicity’ term was a pragmatic choice made due to the low volume of research focusing on different aspects of inequality; it does not reflect that these individual aspects are, in fact, equivalent nor that each individual aspect should not receive consideration in future research. The review focus on gender and race/ethnicity follows Acker’s (2006) theory of ‘inequality regimes’ which holds that relations between members in an organization are not only hierarchically ordered according to occupational positions, e.g. CEO—warehouse manager—warehouse staff, but that these relations also tend to be gendered and racialized.

Research in the goods supply chain outside warehousing shows evidence of inequality in both work organization, work environment conditions, and employment conditions. Studies of work organization in retail stores make visible that certain work tasks and skills are viewed as masculine or feminine and are therefore perceived by employees as being more suitable for men or women, respectively (Johansson and Lundgren, 2015; Johansson, 2016). That the preconceived notions between masculinity and physically demanding work can be propagated through formal task assignment, was shown in a study where women were typically allocated cashier duties while men were given heavy lifting tasks; this also shows that women are assumed to be more suitable than men for front-line service work (Williams, 2004). Inequality in employment conditions has been found in US drayage trucking, i.e. the transporting of cargo containers over short distances between different modes of travel used for the longer transportation legs, e.g. from a port to a rail yard. There, increased contract employments and decreased incomes occurred parallel to the hiring of Latino immigrant workers into the previously ‘white’ occupation. The economic rationales of companies in avoiding employer costs for standard employees related to, e.g. legal responsibilities, clash with the Latino immigrant workers’ access to fair working conditions and could result in experiences of discrimination among the racialized Latino workforce (Bonacich and Wilson, 2008). Similar patterns of racial/ethnic inequality have been seen among seafarers transporting goods (Bonacich and Wilson, 2008), longshore workers loading and unloading goods (Alimahomed-Wilson, 2012), and in-store retail staff selling goods (Storer *et al.*, 2020).

**Figure 1.**
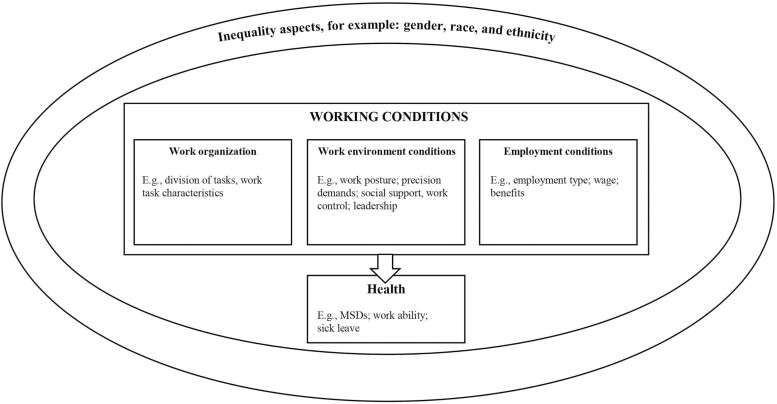
Model illustrating the relationships between concepts used in the present paper.

Figure 1 illustrates the relationship and understanding between working condition aspects, inequality, and health applied in the present review. Here, *working conditions* include work organization, work environment conditions, and employment conditions. We use the term *work organization* to describe the way work is organized at the workplace, including what work tasks are performed, by whom, and how. *Work environment conditions* include both physical factors, for example working postures, workloads and precision demands, and psychosocial factors, e.g. demands, support, control, and leadership. Finally, *employment conditions* refer to the way an employee is employed, including employment type (regular employment at the company or temporary employment via an agency), the extent of work (full-time or part-time), wage levels and benefits. While working conditions presumably differ between geographical regions, the present review has a global scope and considers studies of warehousing irrespective of its geographical context. Figure 1 also illustrates *inequality* as a context within which working conditions can be considered. Worker health is understood as an outcome of the (in)equal working conditions.

No publication has, to our knowledge, systematically reviewed the scientific knowledge of inequality in work organization, work environment, and employment conditions in warehousing. The need for such a review is particularly motivated by the rapid rise of online retailing, and the difference in the goods handled in online retail warehousing compared with traditional warehouses. For example, handling of small packages is more common in online retail warehousing (Boysen *et al.*, 2019) than in traditional warehousing. Perhaps as a result, a larger proportion of women is estimated to work in online retail warehouses than traditional warehouses (Gutelius and Theodore, 2019). Assumptions regarding different capacities between men and women and different racial/ethnic groups of workers, including (im)migrant workers, may influence hiring and task allocation practices in modern retail warehouses and thus current working conditions should be mapped to help guide industry development (cf. Cockburn, 1985; Bonacich and Wilson, 2008; Gutelius, 2016; Wilcox *et al.*, 2022).

Thus, the aim of the present systematic review is to identify, summarize, and discuss research focused on the expressions of inequality and their effects on warehouse working conditions, the latter referring to work organization, work environment conditions, and employment conditions. Inequality aspects are limited to gender and race/ethnicity, the latter referring to race, ethnicity, country of birth, and (im)migration status among employees, as explained above. The review will also identify knowledge gaps and possible areas for future studies.

In addressing this field of research from a broad perspective and including quantitative and qualitative literature, we observe a difference in research endpoint traditions. While some studies deal with the *expressions* of inequality in working conditions, other studies instead focus on the *effects* of inequalities. In general, but not always, studies using qualitative methods investigate inequality as a central part of the study aim, while studies using quantitative methods consider (in)equality as a determinant of the eventual study outcome, e.g. health of men and women, or of different racial/ethnic groups. A comprehensive understanding of inequality and working conditions, and employee health, in warehouses requires studies from both traditions to be addressed.

## Methods

### Search string and electronic search

Literature searches were conducted by the first author using Scopus and Web of Science. The search string (see Supplementary Material A) was designed to find working conditions studies in warehouse settings. The formatting of the search profile was adapted to match the operational requirements of each database.

Inequality-related search terms, such as ‘gender’, ‘race’, or ‘ethnicity’, were not included in the string. This permits inclusion of studies implicitly addressing equality, e.g. studies comparing conditions between men and women whilst not specifically mentioning gender inequality. Contents related to gender and racial/ethnic inequality was therefore identified manually during the screening process.

Our first search was conducted in February 2021. A supplemental search was performed in November 2021 to ensure the review was up to date, covering publications after February 2021 as well as 2022 pre-publications.

### Screening

Title, abstract, and keywords screening of the records resulting from the searches was performed using the Rayyan online review tool, which is a platform for collaborative and systematic sorting of articles (Rayyan, 2022). All records were screened by two reviewers: the first author screened all articles, and the three co-authors were each randomly assigned a third of the records for screening. Conflicts were resolved by discussion until consensus or, if needed, by inviting a third reviewer to finalize the decision. The same approach was utilized in the full-text screening.

For inclusion, articles had to fulfill all of the following criteria: (i) written in English or a Scandinavian language; (ii) published in peer-reviewed journal; (iii) contain original data collected from actual workers (systematic reviews, laboratory studies, protocol papers, and theoretical studies only using secondary data were not included); (iv) occur in warehousing; (v) consider working conditions (i.e. work organization, work environment conditions and/or employment conditions); (vi) address gender and/or racial/ethnic inequality; (vii) provide empirical data, and (viii) consider working conditions and inequality at the workplace level. Rejections were made according to this hierarchical order. Consensus on rejection criteria was required between reviewers for rejection during full-text screening. Only articles meeting all 8 criteria were included.

We deemed studies to have addressed gender or racial/ethnic inequality if they referred explicitly to gender or race/ethnicity in the study aim, findings, or discussion, or implicitly assessed inequality in relation to their findings by using close terms, such as men/male, women/female, Latino/a/x, white/non-white, migrant/immigrant. We therefore included both quantitative studies comparing different groups, e.g. men and women, and qualitative studies discussing, e.g. masculinity as part of their results, even if the authors did not explicitly describe their data as providing evidence of (in)equality.

### Assessment of risk of bias

We evaluated the risk of bias for according to established methods. Qualitative studies were assessed according to a checklist provided by the Swedish Agency for Health Technology Assessment and Assessment of Social Services handbook, covering aspects such as assessment of theoretical and empirical correspondence, methods for data collection and analysis, and the role of the researcher(s) and their relationship to the participants (SBU, 2020). Cross-sectional observational, and longitudinal Cohort and case-control studies were assessed according to checklists provided by the U.S. Department of Health and Human Services National Institutes of Health, including, e.g. formulation of research questions, study and target population, blinding, exposure, and outcome measures and how they are assessed (National Institutes of Health, 2013). Both checklists classified quality in three levels (good, fair, and poor). All articles were assessed by two reviewers independently, and then—in case of diverging ratings—a consensus of ratings was reached through discussion.

### Data extraction

Data were extracted according to a modified list of items as suggested by the Cochrane Handbook for Systematic Reviews of Interventions (Noyes *et al.*, 2021; Tianjing *et al.*, 2021). Summary tables with data corresponding to the aims of the present review were created for qualitative and quantitative study findings.

## Results

### Literature searches

**Figure 2.**
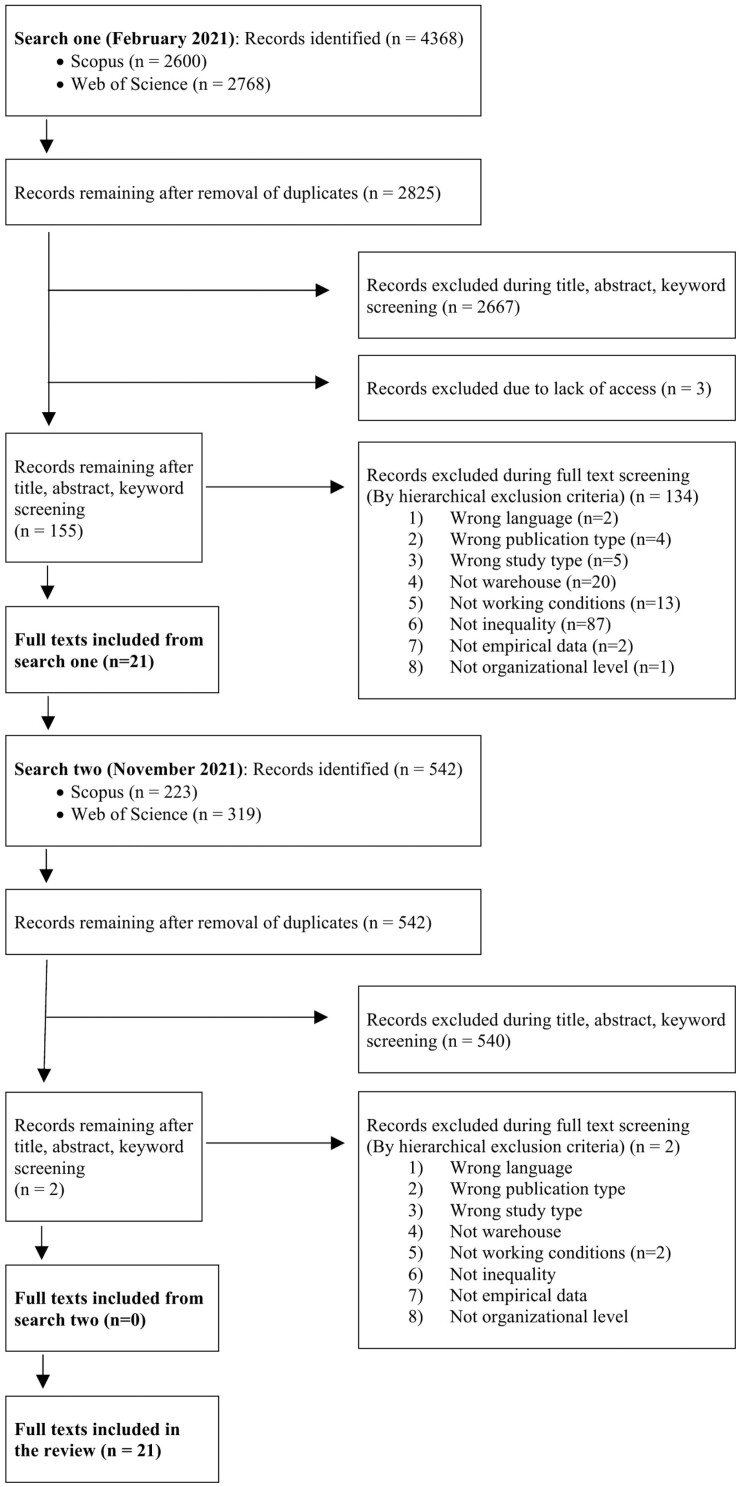
Systematic literature review process summary (Page *et al.*, 2021).

The first search (February 2021) yielded 4368 records, and 2825 articles remained after the removal of duplicates. Screening of title, abstract, and keywords led to exclusion of another 2667 articles. An additional 3 articles were excluded due to lack of access to the full-text PDF, leaving 155 articles for full-text assessment. During the full-text screening, 134 articles were excluded. The supplementary search (November 2021) resulted in 542 records. Screening of title, abstract, and keywords resulted in 2 potentially relevant articles, both of which were excluded after full-text reading. In total, this left us with 21 articles for inclusion. See Fig. 2 for a PRISMA flow chart of the search process and the reasons for exclusion.

### Risk of bias

About 14 studies were rated as having good quality, i.e. a low risk of bias (Szilagyi, 1980; Collinson, 1987; Hoppe *et al.*, 2010, 2014; De Lara *et al.*, 2016; Elliot and Long, 2016; Briken and Taylor, 2018; Kaminer, 2018; Loewen, 2018; Jordhus-Lier *et al.*, 2019; Lindemann and Boyer, 2019; Zampoukos *et al.*, 2018; Gruchmann *et al.*, 2020; Dörflinger *et al.*, 2021). Six studies were rated as having fair quality, i.e. a moderate risk of bias (Katzell *et al.*, 1961; Mars, 2009; Fournier *et al.*, 2017; Allison *et al.*, 2018; Flodin *et al.*, 2018; Alimahomed-Wilson, 2019). One study was rated as having poor quality, i.e. a high risk of bias (Cillo and Pradella, 2018). A common issue among the 15 qualitative studies was a lack of sufficient information about the process of analyzing the empirical material. Among the six quantitative studies, five had a cross-sectional design which dictates a ‘fair’ rating according to the bias evaluation tool, even if this study design is appropriate for the research questions.

### General study characteristics

All studies considered warehouse working conditions and inequality, even if they differed in the way this topic was approached. Tables 1 and 2 provide a comprehensive summary of data from each individual study. Four studies were conducted in wholesaling warehouses, two in furniture distribution centers, and one in each of online retailing-, mail order-, third-party logistics, and drugs- and pharmaceutics warehouses, and a merchandise distribution center. Four studies include two or more warehouse types, and six did not specify the warehouse type. Eight studies addressed gender, eight studies addressed race/ethnicity and five studies addressed both gender and race/ethnicity. One study focused on gender and mentioned immigration and language barriers as potentially related to the low response rate (Flodin *et al.*, 2018). Ten studies were conducted in North America, ten in Europe, and one in Asia. Most of the studies (*n* = 17) were published in 2010 or later.

**Table 1.** Quantitative studies included in the review. In the table, studies addressing race, ethnicity, country of birth, and/or (im)migration status are coded as dealing with race/ethnicity (cf. Hoppe *et al.*, 2010). Studies that do not explicitly use term(s) related to gender or race/ethnicity are coded as dealing with inequality aspects depending on their study focus, and in line with Acker’s (2006) conceptualization of ‘inequality regimes.’ For example, studies focusing on im-/migrants are interpreted as dealing with race/ethnicity (cf. Allison *et al.*, 2018) and studies applying the sex term are coded as dealing with gender (cf. Szilagyi, 1980).

Study^quality^ & country	Warehouse section	Study design & methods	Control variables	Independent variables	Dependent variables	Findings relevant to review aim	Working conditions aspect(s) ^a^	Inequality aspect(s)
Allison, *et al.* (2018) ^Fair^ United States	Retail and supply, 3PL	Observational cross-sectional study Study survey (*n* = 129) and data base survey (*n* = 894)	Age, immigration status (IS), race, educational degree, industry experience, English language ability	Gender, age, marital status, type of warehouse work, employment status (temp or direct hire), average weekly hours (AWH), weeks employed last year (WELY)	Hourly wage (HW), annual income (AI)	In model without correction for type of job: AI negatively associated with being female (gender) and FB (IS). Immigrant women had ↓ HW than non-immigrants. Latino/as had ↓ HW than non-Latino/as. In fully corrected model: NS relationship between HW and gender or IS. Wage differed by job (packing workers earned ↓ than forklift workers) and results suggest job sorting by gender and immigration status: ↑% women in packing than men. ↓% women in forklifting than men. ↑% immigrants in packing than native-born. ↓% immigrants in forklifting than native-born. < % of women than men were employed at least 40 AWH/year. < % of naturalized citizens than native-born and non-citizens were employed at least 40 AWH/year. ↑% naturalized citizens employed directly by warehouse than native-born and non-citizens	Organization, employment	Gender, race/ethnicity
Flodin,*et al.* (2018) ^Fair^ Sweden	Unspecified, forklift operators and office workers	Retrospective cohort study Questionnaire (*n* = 194)	Age, gender	Time in non-neutral neck posture	Self-reported pain in (i) neck and (ii) shoulder	No association between gender and neck pain or shoulder pain among forklift and office workers.	Environment	Gender
Hoppe,*et al.* (2014) ^Good^ United States	Furniture distribution centers	Observational cross-sectional study Questionnaire (*n* = 361), objective measurement	Education, tenure, BMI, smoking	Workplace race/ethnic similarity (R/ES), physical workload (WL), perceived workload (WL), physical exertion (PE), co-worker support (CWS)	Job satisfaction (JSat), lumbar back health (LBH)	Different reactions to R/ES between white and racial/ethnic minority groups: ↑ JSat assoc. with ↑ R/ES in whites; but with ↓ R/ES in Latinos. ↑ LBH assoc. with ↑ R/ES for Latino and African Americans, but with ↓ R/ES for whites. Different associations between racial/ethnic groups: ↓ PE assoc. with ↑ JSat in whites and African Americans, but not Latinos. ↑ CWS assoc. with ↑ JSat among whites and Latinos, but not African Americans. ↑ CWS with ↑ LBH for African American and Latinos but not whites. Whites had ↑ perceived WL, ↓ PE, ↑ W/ES and ↓ LBH than African Americans and Latinos. Latinos had ↑ JSat than whites and African Americans.	Organization, Environment	Race/ethnicity
Hoppe *et al.* (2010) ^Good^ United States	Furniture distribution centers	Observational cross-sectional study Questionnaire (*n* = 471)	Education	Workload (WL), role conflict (RC), role ambiguity (RA), management fairness (MF), social resources (SR), wage fairness (WF)	Job stress (JStr), psychological well-being (PWB)	Different associations with job stress between white and Latino groups: WL and RC assoc. with JStr for Latinos. WL and RA assoc. with JStr for whites. MF neg. assoc. with JStr for Latinos. SR not assoc. with JStr for whites. WF assoc. with JStr among whites, not Latinos. Different associations with psychological well-being between white and Latino groups: RC signific. assoc. with PWB among Latinos. RA signific. assoc. with PWB among whites. SR not assoc. with PWB for Latinos. MF signific. assoc. with PWB for whites. WF assoc. with PWB among whites, not Latinos. Latinos reported ↑ RA, PWB and WF and ↓ JStr than whites. No difference in perceived psychosocial working conditions, Latino workers consistently reported better work-related and general well-being than their White counterparts.	Environment, employment	Race/ethnicity
Katzell, *et al.* (1961) ^Fair^ United States	Drug and pharmaceutical warehouses	Observational cross-sectional study (company as the unit of measurement) Questionnaire, performance measures (72 warehouses, 2520 workers invited to participate)	Size of workforce, city size, wage rate, unionization, percentage male	Job satisfaction (JS) (47 items)	Quantity, quality, profitability, turnover,	↑ JS associated with’ small town culture’ - warehouses located in smaller communities, fewer employees, not being unionized, having ↓ wage rates, and more female than male employees.	Environment, employment	Gender
Szilagyi (1980) ^Good^ United States	Merchandise distribution center	Longitudinal (3 months) case study Questionnaire (*n* = 252 employees (60% F) 22 supervisors (12F/ 10M)	Educational level, job type, seniority level at organization, age, tenure	Perceived leader reward behavior (LRB) of male and female supervisors	Goal attainment (GA), absenteeism, work satisfaction (WS)	No differences in GA, absenteeism, or WS attributed to sex of leader versus subordinate Perceptions of LRB not influenced by sex of leader versus subordinate.	Environment	Gender

^a^‘Organization’ refers to work organization, ‘environment’ to work environment conditions and ‘employment’ to employment conditions.

**Table 2.** Qualitative studies included in the review. In the table, studies addressing race, ethnicity, country of birth and/or (im)migration status are coded as dealing with race/ethnicity (cf. Hoppe *et al.*, 2010). Studies that do not explicitly use term(s) related to gender or race/ethnicity are coded as dealing with inequality aspects depending on their study focus, and in line with Acker’s (2006) conceptualization of ‘inequality regimes.’ For example, studies focusing on im-/migrants are interpreted as dealing with race/ethnicity (cf. Allison *et al.*, 2018) and studies applying the sex term are coded as dealing with gender (cf. Szilagyi, 1980).

Study^quality ^& country	Warehouse section	Study design & methods	Findings relevant to review aim	Working conditions aspect(s) ^a^	Inequality aspect(s)
Alimahomed-Wilson, 2019)^Fair^ United States	Unspecified	Observational study Primary and secondary data, the former collected via interviews	The racialization of workers in South California warehouse sector is assoc. with worsening of working conditions, e.g. through temporary agency employment and low salaries. Women workers of color are the most subordinated therein.	Employment	Gender, race/ethnicity
Briken & Taylor (2018) ^Good^ United Kingdom	Online retail	Observational study Interviews	Temporary employment via agencies is common and temps in particular experience issues with employers monitoring and controlling their work. Migrant workers experience discrimination therein.	Environment, employment	Race/ethnicity
Cillo & Pradella (2018) ^Poor^ Italy	Unspecified	Observational study Participant observations, textual analysis	Immigrant workers are victims to exploitative working conditions, including on-call scheduling and unregulated payments, and risk of harsh opposition if speaking up about their situation.	Environment, employment	Race/ethnicity
Collinson (1987) ^Good^ United Kingdom	Mail order	Observational study Interviews, observations	Women are used as a ‘reserve army’ of labor to ensure flexibility for the employer. Women are mostly assigned picking and packing tasks, men often get to do the truck driving and heavy lifting activities.	Organization	Gender
De Lara, *et al.* (2016) ^Good^ United States	Unspecified	Observational study Participant observations, interviews, volunteer work, informal conversations, media reviews	Workers, who are mostly Latino and many undocumented migrants, suffer from insufficient working conditions including low wages and a lack of benefits.	Employment	Race/ethnicity
Dörflinger, *et al.* (2021) ^Good^ Germany + the Netherlands + Belgium	3PL	Observational study Primary and secondary data, the former collected via interviews and observations	Employers’ strategies to control labor production vary geographically depending on political and cultural context. One strategy is to hire agency workers, whose vulnerable position is further strained by the majority being migrants. Tensions between migrants and non-migrants due to different employment statuses and language barriers result in a competitive workplace culture.	Environment, employment	Race/ethnicity
Elliot & Long (2016) ^Good^ United States	Wholesale	Observational study (Covert) participant observations	The employer’s technological monitoring of employee performance gives rise to a masculine culture of competition in the workplace.	Environment	Gender
Fournier,*et al.* (2017) ^Fair^ France + Belgium	Wholesale	Observational study Statistics, archives, interviews, observations	Part-time employment with irregular working hours and low salaries are common, and often assigned to women. Men tend to do the unpacking of incoming goods, women the packing of products for delivery. Automated warehouses are generally staffed by women doing the manual work. Previously, women could be promoted from manual warehouse work to administrative positions; these possibilities have decreased over the years.	Organization, employment	Gender
Gruchmann, *et al.* (2020) ^Good^ Germany	Wholesale, manufacturing	Observational study Interviews, questionnaire, workshop	In one warehouse, men are often assigned the tasks considered by the employer to be physically strenuous; employees do not share the perception of these tasks as demanding. Another employer applies automation technologies to minimize physically demanding tasks, arguing it will make it possible for employees of all genders to perform all tasks. Language barriers between workers make it difficult to raise awareness about issues related to the physiological load of work tasks.	Organization, environment	Gender, race/ethnicity
Jordhus-Lier, *et al.* (2019) ^Good^ Norway	Wholesale, construction	Observational study Interviews, focus group	Temporary employed agency workers are expected to perform better than permanent employees. That many agency workers are Swedes comes with further assumptions of them being hardworking. To get permanent employment, they must keep a good relationship with their employer; the move from Sweden to Norway is seen as the first sign of their assumed high ambitions and work ethic. Different migrant groups generally do different tasks in the warehouse; pickers and packers are Swedish, truck drivers from Eastern Europe.	Organization, employment	Race/ethnicity
Kaminer, (2018) ^Good^ Israel	Wholesale	Observational study (Partly covert) participant observations	The workforce consists mostly of female and immigrant workers; power relations position certain employee groups in a subordinated position. Women, and particularly immigrant women, have limited possibilities to improve the precarious situation. Work is divided into ‘girls’ and ‘guys’ tasks; women unpack boxes and put products into bins, men stack the bins and assist other people with their tasks. Among temporary employees, women earn less than men.	Organization, environment, employment	Gender, race/ethnicity
Lindemann & Boyer (2019) ^Good^ United States	Unspecified	Observational study Focus groups	‘Perma-temp’ term highlights that temporary employment tends to be permanent in its being. Perma-temps precarious situation is intertwined with their gendered, racialized, and classified subordinated position. Women, and more often racialized women, experience sexual harassment and sexism, e.g, women who are perceived to be more good-looking get the easier tasks. Unequal treatment is also related to race/ethnicity. Perma-temp status makes it difficult to raise concerns, given the fear of losing the job. Men are assigned lifting tasks and operating the machinery. Women more often work at the assembly line. Men and women have different salaries not only when tasks are gendered, but also in warehouses where they do the same job.	Organization, environment, employment	Gender, race/ethnicity
Loewen (2018) ^Good^ United States	Wholesale, online retail	Observational study Participant observations, interviews	Men are driving forklifts and operating the machinery. Women are often assigned tasks that are considered physically ‘light’ such as manual picking. The feminized tasks are common in e-commerce warehouses. Gendered division of tasks risks resulting in unequal payment; women do jobs that are paying less.	Organization, employment	Gender
Mars (2009) ^Fair^ England	Wholesale	Case study Qualitative methods with input from survey	A gendered division of work between those working in the warehouse of the company and the office, with the group of office workers being mostly women. Men often works in the warehouse with picking, driving, and un-/loading trucks.	Organization	Gender
Zampoukos, *et al.* (2018) ^Good^ Norway	Unspecified	Observational study Focus groups, interviews	Adapting to the idea of Swedes as hard-working is a way for Swedish migrant workers to get a stable job. Initially, many are temporary employed. Working hard—harder than Norwegians—becomes a strategy to hopefully get permanent employment; this is expressed in working instead of staying at home when sick, among other things. Employers’ technological monitoring of employees and bonus systems reinforce the competitive culture.	Environment employment	Race/ethnicity

^a^‘Organization’ refers to work organization, ‘environment’ to work environment conditions and ‘employment’ to employment conditions.

### Gender

A gendered organization of work was reported in eight studies (Collinson, 1987; Mars, 2009; Fournier *et al.*, 2017; Allison *et al.*, 2018; Kaminer, 2018; Loewen, 2018; Lindemann and Boyer, 2019; Gruchmann *et al.*, 2020). Studies showed men were commonly assigned tasks involving machinery operation (Loewen, 2018; Lindemann and Boyer, 2019) and truck- and forklift driving (Collinson, 1987; Mars, 2009; Allison *et al.*, 2018; Loewen, 2018), while women performed order picking (Collinson, 1987; Loewen, 2018) and packing (Collinson, 1987; Allison *et al.*, 2018). One study showed men were assigned tasks considered by the employer to be the most physically demanding (Gruchmann *et al.*, 2020). One study considered work environment conditions via measurements of the biomechanical workload, but the study did not find an association between gender and neck pain or shoulder pain among forklift operators (Flodin *et al.*, 2018). Regarding employment conditions, wage disparities were reported in five of the papers (Fournier *et al.*, 2017; Allison *et al.*, 2018; Kaminer, 2018; Loewen, 2018; Lindemann and Boyer, 2019), which could be explained by the gendered division of work (Loewen, 2018). However, evidence was also found for men earning more than women when performing the same job (Lindemann and Boyer, 2019).

### Race/ethnicity

Evidence for inequality in the work organization was shown in the division of work tasks along lines of employee (im)migration status and birth country. A Norwegian study demonstrated that workers in picking and packing were often Swedish, whilst truck drivers were migrants from Eastern Europe (Jordhus-Lier *et al.*, 2019). A US study similarly proved that a higher percentage of immigrant workers were employed in packing, and a lower percentage in forklifting, as compared to native-born workers. Workers in packing earned less than forklift drivers (Allison *et al.*, 2018). Regarding work environment conditions, one study showed a connection between workplace racial/ethnic similarity and health: Latino and African American workers in mixed race/ethnic workplaces had worse lumbar back health than workers in homogeneous racial/ethnic workplaces (Hoppe *et al.*, 2014). Evidence for inequalities in employment conditions was presented in terms of income disparities between groups of employees in two studies that found Latino/a workers earned less non-Latinos/as (Hoppe *et al.*, 2010; Allison *et al.*, 2018). One of these studies found Latino workers nevertheless experienced their comparatively low wages to be more fair than the more highly paid White workers perceived their wages (Hoppe *et al.*, 2010). Two studies reported low wage levels among migrant and racialized workers (De Lara *et al.*, 2016; Alimahomed-Wilson, 2019). Inequalities concerning employment conditions were also evident in that immigrant and racialized warehouse workers were commonly victims of precarious employment through ­temporary work agencies (Allison *et al.*, 2018; Zampoukos *et al.*, 2018; Alimahomed-Wilson, 2019; Jordhus-Lier *et al.*, 2019; Lindemann and Boyer, 2019; Dörflinger *et al.*, 2021). Employment differences between immigrant and native employees were shown to lead to competitive cultures at the workplace level (Dörflinger *et al.*, 2021) and increased performance pressure on the temporary employees (Zampoukos *et al.*, 2018; Jordhus-Lier *et al.*, 2019).

### Gender and race/ethnicity

Evidence was found for differences in intersections of gender *and* race/ethnicity related to working conditions. US and Israeli studies showed that the two inequality aspects interact and result in immigrant women (Allison *et al.*, 2018; Kaminer, 2018), Latina women (Lindemann and Boyer, 2019), and women of color (Alimahomed-Wilson, 2019) being subordinated workers. Their subordinated position was evident both in employment conditions, as immigrant women earned less than non-immigrant men and women (Allison *et al.*, 2018), and in the work environment conditions, as immigrant women (Kaminer, 2018) and Latina workers (Lindemann and Boyer, 2019) had limited opportunities to influence the work situation. Additionally, one study addressed gender and language differences between groups of employees and showed that the language barriers make it difficult for employees to raise awareness about issues related to the physiological load of work tasks, but without explicitly linking the two aspects of gender and race/ethnicity together (Gruchmann *et al.*, 2020).

## Discussion

This review aimed to summarize research on gender and race/ethnicity in jobs comprising the warehousing goods handling chain, including both gender and racial/ethnic inequality at work and its significance for working conditions. We found 21 articles that brought attention to the expressions of one or more of these inequality aspects, and the effects on warehouse working conditions, i.e. work organization, work environment, and/or employment conditions.

### Gender

We found evidence of inequality in work organization in terms of division of tasks along lines of gender. We also found that such divisions related to preconceived notions of both the character of the work tasks and of men’s and women’s abilities to perform those tasks. Of the 13 included articles addressing gender, eight demonstrated a gendered organization of work (Collinson, 1987; Mars, 2009; Fournier *et al.*, 2017; Allison *et al.*, 2018; Kaminer, 2018; Loewen, 2018; Lindemann and Boyer, 2019; Gruchmann *et al.*, 2020). Evidence of men being assigned tasks considered by the employer to be the most physically demanding (Gruchmann *et al.*, 2020) and women getting the tasks considered physically ‘light’ (Loewen, 2018) explains, at least in part, findings from warehouse studies that the gendered work organization was associated with differences in the assumed physical load of tasks (cf. Cockburn, 1985; Gutelius, 2016). One of the included studies conducted in an online retail warehouse argued that ‘women are segmented into lower paying *light* manual labor, such as the picking processes that are core to e-commerce operations’ (Loewen, 2018: 707–708), which agrees with notions that US online retailing will likely hire a larger proportion of women to order picking than traditional warehouses (Gutelius and Theodore, 2019), and that smaller packages are likely handled in online retail than in traditional warehouse work (Boysen *et al.*, 2019). Considering that the online retail studies are all from the USA, these predictions may not be applicable to geographical areas with a different labor politics and societal structures with other implications on equality.

One of the articles in the review showed that a warehouse employer introduced automation technologies with the argument that it would allow both women and men to do the work (Gruchmann *et al.*, 2020), which can be interpreted as a belief of assisting work technology being a tool to equalize working conditions. However, this may not be the case as technology is commonly seen as a domain for men (Cockburn, 1985), and the introduction of technology could possibly lead to further exclusion of women from the work. In keeping with this, the present findings show men more commonly assigned machine operation (Loewen, 2018; Lindemann and Boyer, 2019) and truck- and forklift driving (Collinson, 1987; Mars, 2009; Allison *et al.*, 2018; Loewen, 2018). Further considering that women were often doing the order picking (Collinson, 1987; Loewen, 2018) and packing (Collinson, 1987; Allison *et al.*, 2018), it is reasonable to assume that the division of tasks in warehouse work along lines of gender likely results in different patterns of MSDs for men and women. Still, we do not know from the present findings how gender inequality in work organization and work environment conditions in warehouses influences health outcomes (cf. Flodin *et al.*, 2018).

### Race/ethnicity

The review results suggest that the shaping of inequality in warehousing working conditions relate to complex hierarchies of workers based on assumptions about specific nationalities and geographical regions. Not only does the results show task division between immigrant- and native-born workers (Allison *et al.*, 2018), but there is likewise evidence of different migrant groups doing different tasks in warehousing; a study in a Norwegian warehouse found Swedes often performed picking and packing, while Eastern Europeans performed truck driving (Jordhus-Lier *et al.*, 2019). The Norwegian study context further shows that preconceived ideas of the abilities of certain groups could affect not only the work organization, but also the work environment conditions: Swedish workers in Norway were perceived as hardworking. Even if they were not looked upon in a condemning way, such prejudices could still have negative outcomes on health. For example, Swedes mentioned that they worked instead of staying at home when sick (Zampoukos *et al.*, 2018). Health outcomes related to the employee composition of racial/ethnic backgrounds were also seen in the US context, as Latino and African American workers in mixed race/ethnic workplaces had worse lumbar back health than workers in homogeneous racial/ethnic workplaces. One reason could be that in the mixed workplaces the Latino and African American workers must work harder than whites to improve their subordinate position (Hoppe *et al.*, 2014). Thus, the research suggests that differences in workload and health among workers in warehousing are caused by factors in the work organization (Braam *et al.*, 1996), which need to be investigated in a racial/ethnic context.

We have used the ‘race/ethnicity’ term in the present review to describe studies assessing racial and ethnic groups of warehouse workers—e.g. white, Latino, and African American workers in US warehouses (Hoppe *et al.*, 2014) – as well as (im)migrant groups, such as Swedish workers in Norwegian warehouses (Zampoukos *et al.*, 2018; Jordhus-Lier *et al.*, 2019). A geographical distinction seems evident: most studies referring to ‘race’ and/or ‘ethnicity’ were conducted in the USA (Hoppe *et al.*, 2010, 2014; Alimahomed-Wilson, 2019; Lindemann and Boyer, 2019), while European studies focused more often on (im)migrants (Zampoukos *et al.*, 2018; Jordhus-Lier *et al.*, 2019; Dörflinger *et al.*, 2021) and less often on race and ethnicity (Briken and Taylor, 2018; Cillo and Pradella, 2018). Additionally, one German study, labeled by us as a study of ‘race/ethnicity,’ did not explicitly consider race, ethnicity or (im)migrant groups of workers, but instead compared groups of workers by German language proficiency, which could then be interpreted as a comparison of native-born and (im)migrant warehouse workers (Gruchmann *et al.*, 2020). One possible explanation to the different approaches, and the limited number of European studies dealing explicitly with ‘race’ or ‘ethnicity’, is the controversy and difficulties of investigating race in certain contexts. Nordic scholars have argued that the taboo surrounding ‘race’ in the Nordics could partially be explained by a shame regarding the historical involvement of the Nordic countries in race biology, and the ongoing and societal ignorance around the same, together with the current national self-image of the Nordic countries being at the forefront of equality (Hübinette and Mählck, 2015; Rastas, 2019). We deemed that the different terminologies should be taken into consideration together as possibilities for generalization between study contexts were high.

### Gender and race/ethnicity

Evidence of complexity in the shaping of inequality in warehousing working conditions is also the most important contribution from the studies addressing intersections of gender *and* race/ethnicity. Immigrant women (Allison *et al.*, 2018; Kaminer, 2018), Latina women (Lindemann and Boyer, 2019), and women of color (Alimahomed-Wilson, 2019) were found to be subordinate workers in warehousing, in accordance with Acker’s (2006) theory that the relations between members in an organization are not only hierarchically ordered according to occupational positions, but that these relations also tend to be gendered and racialized. The embeddedness of gender and race/ethnicity in organizations means that the specific shape and effects of inequalities are difficult to reduce to one single aspect alone (Acker, 2006). The study showing in their uncorrected model that immigrant women had lower hourly wages than non-immigrant men and women (Allison *et al.*, 2018) suggests that immigrant women are not only lower on the hierarchy due to their immigrant status but also because they are female. While evidence was found for a hierarchy based on gender and racialization and its association with employment conditions, little is known about the possible implications of hierarchical relations on occupational health in warehoused goods handling.

### Suggestions for future research

First, we found evidence of inequality based on gender and race/ethnicity in both work organization [gender (Collinson, 1987; Mars, 2009; Fournier *et al.*, 2017; Allison *et al.*, 2018; Kaminer, 2018; Loewen, 2018; Lindemann and Boyer, 2019; Gruchmann *et al.*, 2020); race/ethnicity (Hoppe *et al.*, 2014; Allison *et al.*, 2018; Jordhus-Lier *et al.*, 2019)], work environment conditions [gender (Katzell *et al.*, 1961; Szilagyi, 1980; Elliot and Long, 2016; Flodin *et al.*, 2018; Kaminer, 2018; Lindemann and Boyer, 2019; Gruchmann *et al.*, 2020); race/ethnicity (Hoppe *et al.*, 2010, 2014; Briken and Taylor, 2018; Cillo and Pradella, 2018; Kaminer, 2018; Zampoukos *et al.*, 2018; Lindemann and Boyer, 2019; Gruchmann *et al.*, 2020; Dörflinger *et al.*, 2021) and employment conditions (gender (Katzell *et al.*, 1961; Fournier *et al.*, 2017; Allison *et al.*, 2018; Kaminer, 2018; Loewen, 2018; Lindemann and Boyer, 2019); race/ethnicity (Hoppe *et al.*, 2010; De Lara *et al.*, 2016; Allison *et al.*, 2018; Briken and Taylor, 2018; Cillo and Pradella, 2018; Zampoukos *et al.*, 2018; Alimahomed-Wilson, 2019; Jordhus-Lier *et al.*, 2019; Dörflinger *et al.*, 2021)]. Yet, despite scholars’ emphasis on the intertwined nature of gender and race/ethnicity in the study of inequality at the workplace level (Holvino, 2001; Acker, 2006), research addressing intersections of gender *and* race/ethnicity in warehousing is scarce (Allison *et al.*, 2018; Kaminer, 2018; Alimahomed-Wilson, 2019; Lindemann and Boyer, 2019; Gruchmann *et al.*, 2020). This underlines the importance of considering gender and race/ethnicity as mutually constitutive factors in the shaping and effects of inequal working conditions.

Second, we identified 15 studies using a qualitative approach to investigate ways that gender and race/ethnicity play a role in the structure and organization of warehouse work. Only six studies considered, however, how (in)equality relates to working conditions in quantitative terms, and only four of those were conducted in 2010 or later (Hoppe *et al.*, 2010, 2014; Allison *et al.*, 2018; Flodin *et al.*, 2018). The limited number of recent studies with a quantitative approach to gender and race/ethnicity is remarkable, since certain issues can best be answered using quantitative data. Does biomechanical workload differ between men and women performing MMH? Does temporary employment influence the occurrence of MSDs between/within different ethnic/racial groups? In acknowledging the ­importance of studying both the expression and effects of inequality, future quantitative studies should consider *how* inequality impacts on warehouse working conditions and thus the health of employees, in addition to qualitative studies focusing on *why* inequality occurs.

Third, we encourage studies in the growing online retail section of warehousing. Considering that the gendered division of warehouse work tasks could be partly explained by assumed differences in the physical load of tasks, and notions of women being less capable to perform ‘heavy’ work (Gruchmann, *et al.*, 2020; cf. Cockburn, 1985; Gutelius, 2016), it would be relevant to explore tasks division by groups in online retail warehouses and its possible effects on individual workers. That online retailing involves small package handling to a relatively large extent (Boysen *et al.*, 2019) makes it likely that work will comprise more MMH and less movement of goods with trucks and forklifts than in other sections of warehousing. If men in warehouse work commonly drive trucks and forklifts (Collinson, 1987; Mars, 2009; Allison *et al.*, 2018; Loewen, 2018), what tasks will be assigned men and women in online retail warehouses?

### Strengths and limitations

Our limitation to only address gender and race/ethnicity is motivated by Acker’s (2006) argument that ­relations between employers and employees in a workplace are not only hierarchically ordered according to occupational positions but tend also to be gendered and racialized. However, we do not claim that gender and race/ethnicity are the only inequality aspects relevant to warehouse work; while hierarchies of gender and race/ethnicity are occurring most often, the base of inequality may vary between different workplaces (Acker, 2006). The literature searches suggested considerations of inequality based on age (Gekara *et al.*, 2019) and disability status (Houtenville and Kalargyrou, 2015; Moore *et al.*, 2020) may also be relevant in warehousing.

We view our decision to include studies that both explicitly and implicitly focused on (in)equality to be a strength of this review. This led to the inclusion of more articles than we would have otherwise found. Another strength lies in our multidisciplinary approach and the inclusion of both qualitative and quantitative studies. Not limiting our scope to occupational health publications, but also allowing for research from academic fields with different endpoint traditions resulted in a comprehensive overview of previous research on warehouse working conditions. That most of the studies had a low risk of bias further substantiates the usefulness of our findings.

### Concluding remarks

Through identifying, summarizing, and discussing research focused on expressions of (in)equality and their effects on warehouse working conditions, we found evidence that gender and race/ethnicity influence work organization, work environment conditions, and employment conditions. The findings also highlight a hierarchy of inequalities based on intersections between gender and race/ethnicity, whereby racialized and (im)migrant women are positioned at the bottom of the hierarchy.

The review points to the need to consider how expressions and effects of inequality in warehousing relates to the constructs of gendered and/or racialized symbols and images of both the work and the workers, and how these two constructs interact at an organizational level. To improve warehouse working conditions and subsequently occupational health, including the prevention of MSDs, both researchers and policy makers need to take into consideration explicit and tangible, as well as implicit and intangible, expressions of inequality. This is especially relevant to the growing warehouse section of online retailing.

## Supplementary Material

wxac098_suppl_Supplementary_Material

## Data Availability

*No new data were generated or analysed in support of this research.*
