# Supplementary material for: A Systematic Review of Work Organization, Work Environment, and Employment Conditions in Warehousing in Relation to Gender and Race/Ethnicity
Source: Ann Work Expo Health. 2023 Jan 30;67(4):430–47. doi: 10.1093/annweh/wxac098 (PMC10119697; doi:10.1093/annweh/wxac098)
Supplement: wxac098_suppl_Supplementary_Material [file wxac098_suppl_supplementary_material.docx]

A Systematic Review of Work Organization, Work Environment and Employment Conditions in Warehousing in Relation to Gender and Race/Ethnicity

Klara Rydström, Jennie Jackson, Kristina Johansson, Svend Erik Mathiassen

**Supplementary material A.** Search string

(warehous*  OR {logistics sector}  OR {logistics industry} OR {distribution center}  OR  {distribution centers}   OR {fufillment centre}  OR  {fulfillment centers}  OR  {packing center} OR {packing centers}  OR  {packing facility} OR {packing facilities}  OR {distribution facility} OR {distribution facilities})

AND

(work OR labor OR {work environment} OR {working environment} OR {work conditions} OR {working conditions} OR organization OR {job satisfaction} OR {work satisfaction} OR workload OR workplace)

AND NOT

({data warehouse} OR {data warehousing} OR {data mining} OR {data-mining} OR {fuzzy})
